# The treatment of posterolateral tibial plateau fracture with a newly designed anatomical plate via the trans-supra-fibular head approach: preliminary outcomes

**DOI:** 10.1186/s12891-021-04684-w

**Published:** 2021-09-18

**Authors:** Pan Cai, Mingyuan Yuan, Houlin Ji, Xu Cui, Chao Shen, Xiaoxiao Zhou, Yang Yang

**Affiliations:** 1 Department of Orthopedics, Shanghai University of Medicine & Health Sciences Affiliated Zhoupu Hospital, 1500 Zhouyuan Road, Pudong New District, Shanghai, China; 2 Department of Radiology, Shanghai University of Medicine & Health Sciences Affiliated Zhoupu Hospital, Shanghai, China; 3 Graduate school of Shanghai, University of Traditional Chinese Medicine, Shanghai, China; 4 Department of Orthopedics, Taizhou Hospital of Zhejiang Province, Affiliated to Wenzhou Medical University, No. 150 Ximen Street, Zhejiang 317000 Linhai , China

**Keywords:** Posterolateral Tibial Plateau Fracture, Anatomical Plate, Trans-supra-fibular Head Approach, Outcomes

## Abstract

**Background:**

There are no ideal plates or approaches for anatomical restoration and rigid fixation of posterolateral tibial plateau fractures. This study aimed to evaluate the short-term preliminary outcomes of our novel anatomical plate placed via the trans-supra-fibular approach to treat posterolateral tibial plateau fractures.

**Methods:**

From May 2016 to May 2018, 23 consecutive patients with posterolateral tibial quadrant fractures underwent open reduction with internal fixation via the trans-supra-fibular-head approach with our newly developed plate. The tibial plateau-tibial shaft angle (TPTSA), lateral posterior tibial slope angle (LPSTA), step-off, and condylar widening were measured on radiological images pre-operatively, 3 days post-operatively, 3 months post-operatively, and at the final follow-up examination. The radiological Rasmussen score was calculated, and the Hospital for Special Surgery (HSS) knee score was assessed to evaluate the functional outcomes.

**Results:**

The LTPSA, TPTSA, step-off, and condylar widening at 3 days post-operatively, 3 months post-operatively, and at the final follow-up were significantly different (*p* = 0.001) compared with those pre-operatively, as was the radiological Rasmussen score (*p* = 0.001). The HSS score at the final follow-up was 89.10 ± 5.94 (range, 78–98), which was significantly higher than that at the 3-month follow-up 84.36 ± 6.76 (range, 74–96); *p* = 0.001).

**Conclusions:**

Our newly designed anatomical plate placed via the trans-supra-fibular approach can effectively treat posterolateral tibial plateau fractures. We noted minor trauma, stable fixation, and satisfactory clinical results.

## Background

**Fig. 1:**
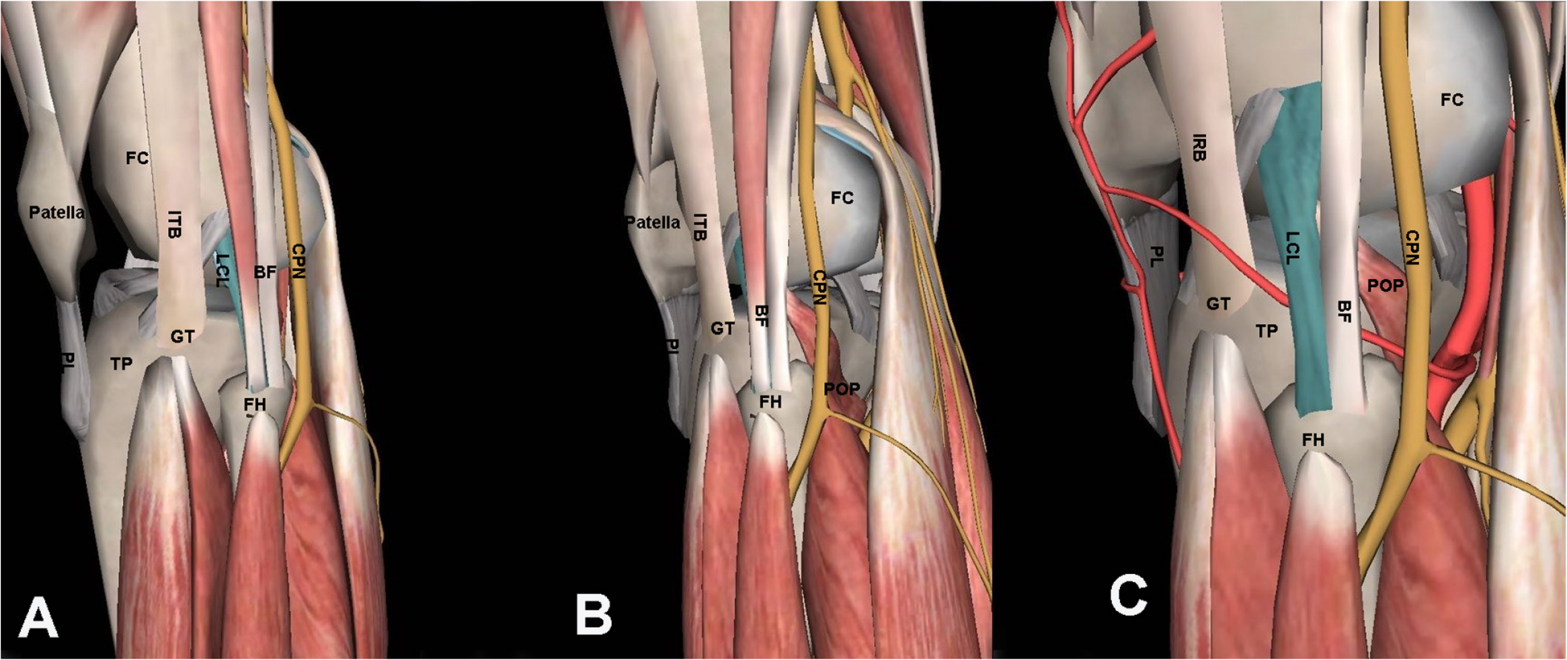
Three-dimensional schematic diagram showing the anatomical structures around the posterolateral tibial plateau. Lateral (**A**) and posterolateral (**B**) views illustrate the anatomical relationship. The LCL can be observed after removing the biceps femoris short head under the biceps femoris (**C**). FH, fibular head; TT, tibial tubercle; GT, Gerdy’s tubercle; FH, fibular head; ITB, iliotibial tract; BT, biceps tendon; FC, femoral condyle; TP, tibial plateau; BF, biceps femoris; LCL, lateral collateral ligament; POP, popliteus; PL, patellar ligament; CPN, common peroneal nerve

Articular malreductions can reach > 30 % after tibial plateau fracture fixation [1], and complex tibial plateau fracture management remains clinically challenging. Fractures of the posterolateral quadrant of the tibial plateau are common and are among the most difficult to treat, while the optimal treatment remains controversial. Visualizing and manipulating the posterolateral fracture fragments via the common anterolateral approach are typically obstructed by the fibular head, lateral collateral ligament, and posterolateral corner complex structure (Fig. 1 A-C). These have led to multiple surgical approaches aimed at improving the visibility of and access to the region. These include femoral epicondyle osteotomy, fibular resection osteotomy, digastric fibular head osteotomy, and posterolateral and posterior approaches [2]. Posterior and posterolateral approaches allow direct exposure of the fractured fragment of this specific portion of the tibial plateau and the application of an anti-glide or buttress plate for fixation [3–5]. However, these approaches that involve intricate anatomical structures are disadvantageous because they involve a high risk of iatrogenic injury to local vasculatures and the common peroneal nerve, thereby presenting a difficult challenge to the treating surgeons.

Management is technically demanding for an orthopedic surgeon because restoration of the lower extremity mechanical axis, anatomical reduction of the articular surface, and achievement of sufficient post-operative ligamentous stability and rigid internal fixation of fractured fragments are necessary to allow for early movement [4]. Various plate systems have been described, such as the specially designed plate placed under proximal tibiofibular joint for posterolateral tibial plateau fractures [6], in the posterolateral proximal tibial plateau [7], in the posterior proximal tibia, the new WAVE plate owned horizontal epiphyseal arm for posteromedial and posterolateral [8]. Even with locking screws, front-to-back screws, including ‘magic screws,’ have a mechanical disadvantage in terms of cantilever loading [9]. The treatment of posterolateral tibial plateau fractures via the anterolateral supra-fibular-head approach or modified anterolateral approach has been introduced [10–12]. These methods are claimed to have advantages, including ease of operation, less trauma, and good clinical outcomes. However, the current plate technology may not adequately allow proximal positioning of the subchondral screws (raft screws) to support posterolateral joint surface impaction injuries. However, whether a lateral locking plate can provide sufficient stability to the posterolateral fragments remains controversial, and lesser locking screws are unavailable to firmly hold the posterolateral fragments, which may lead to fixation failure in this area [13]. A combined lateral approach with a limited posteromedial approach using a pre-contoured one-third tubular ‘hoop-plating’ plate was described [14]. With that approach, the plate was slid horizontally and wrapped under tension around the posteromedial and posterolateral corners of the tibial plateau, which provided support, compression of the proximal tibia, and circumferential containment of the posterior articular fracture and periarticular rim fragments that are held in place by the implant. Another study described a ‘reverse L-shaped’ posterior approach that allows access and more distal fixation posteriorly using a pre-contoured buttress or anti-glide plate for fixation [15]. An extended anterolateral approach to such fractures with an LCP or Pilon plate provides a buttress, maintains articular surface reduction, and provides resistance against local depression loads [16]. However, these internal fixation instruments were not specifically designed primarily for the posterolateral tibial plateau, and most plates need to be pre-contoured so that the fragments can be held in place by more screws [5, 10, 13]. If ≤ 1 screw crosses the posterolateral fragments, the results could be compromised. Therefore, we designed a special anatomical locking plate for these types of fractures. In this study, we investigated the clinical and radiological outcomes of our novel anatomical plate placed via the trans-supra-fibular-head approach to treat posterolateral tibial plateau compressed fractures.

## Materials and methods

From May 2016 to May 2018, this prospective study analyzed 23 consecutive patients (14 men and 9 women; average age, 53.43 ± 14.53 years, 29.00–79.00 years) diagnosed with posterolateral tibial quadrant fractures who underwent open reduction with internal fixation via trans-supra-fibular-head approach using our novel designed lateral anatomical buttress plate at our institution (Table 1). The inclusion criteria were as follows: posterolateral tibial plateau fracture as revealed by computed tomography (CT) examination; treatment with open reduction and internal fixation via the trans-supra-fibular-head approach using our plate; and completion of all post-operative follow-up examinations. Patients with a step-off of < 3 mm in a lateral tibial plateau fracture [17], fractures with established compartment syndrome, and pathological fractures were excluded. Surgical procedures of all patients were performed by the same senior orthopedic surgeon. Radiographic images of the anteroposterior and lateral views and three-dimensional CT reconstruction images at different follow-up time points were reviewed independently by two authors. The trauma mechanism included traffic accidents (9 cases) and falls from height (14 cases). All tibial plateau fractures in this study were classified according to Schatzker’s classification [18] and the Orthopaedic Trauma Association Classification [19] (Table 1). This study was approved by the institutional review board of the corresponding author’s institution, and informed consent was obtained from all patients.

**Table 1:** Summary of the Main Demographic Data

Basic characteristics	
**Total patients**	23
**Male/female**	14/9
**Left/right Knee**	14/9
**Age (years) (range)**	53.43 ± 14.53 (29.00–79.00)
**Follow-up times (months) (range)**	28.17 ± 7.13 (17–44)
**Injury (traffic accident/fall)**	9/14
**Time of fracture union (weeks) (range)**	12.13 ± 1.36 (9–15)
**AO type (B/C)**	18/5
**Schazter’ type (II/III/V/VI)**	10/5/2/6

### Surgical Technique

#### Preparation and exposure

**Fig. 2:**
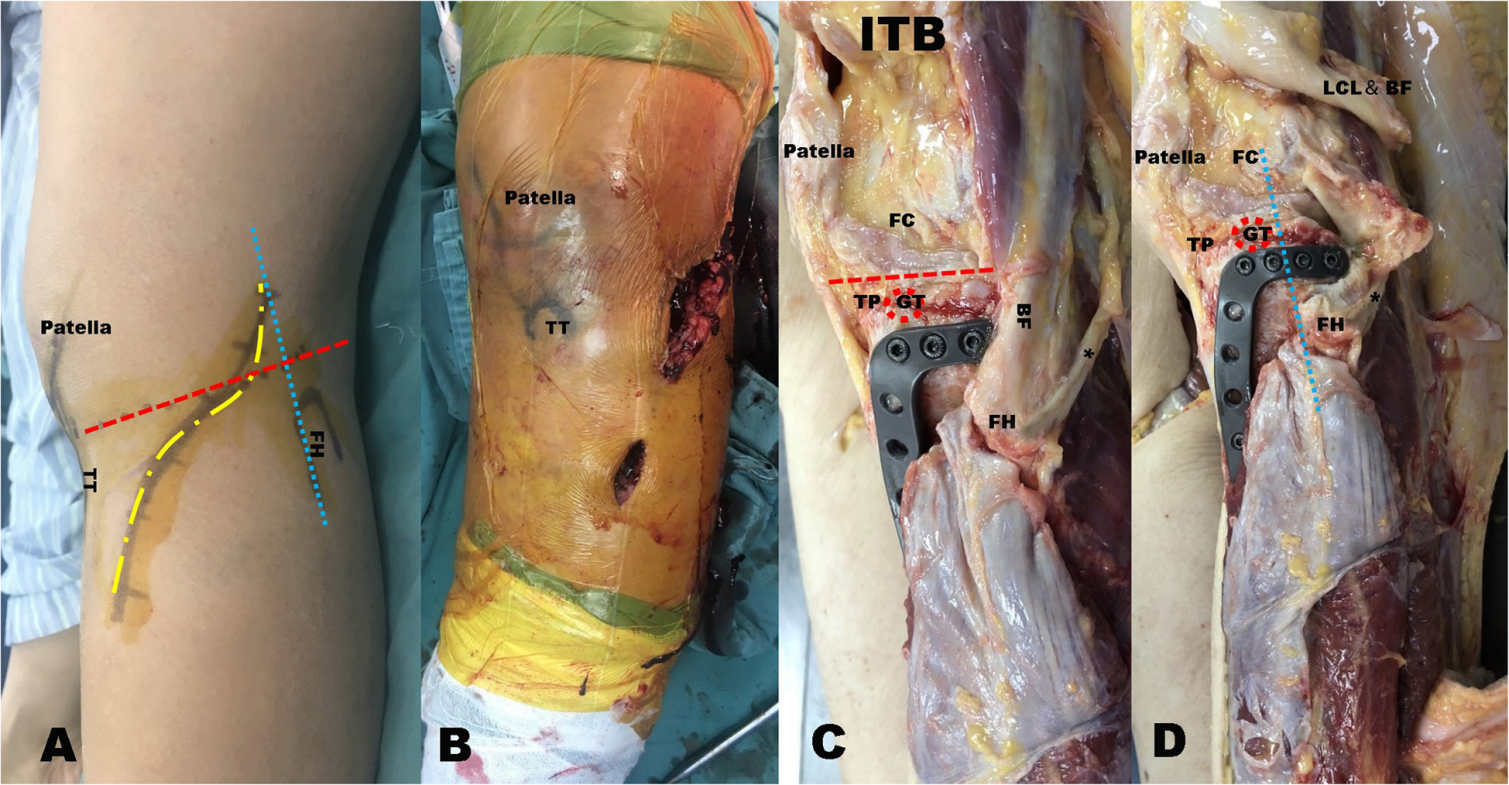
**A**. Location of the incision and anatomic landmarks. The red dashed line represents the joint line, the blue dotted line represents the front edge line of the fibular head, and the yellow dotted line represents the surgical incision. **B**. Surgical incision. **C** and **D**, Fresh specimen autopsy showing the anatomic structures around the posterolateral tibial plateaus, and simulated implantation of the novel plate and screw, both GT had been detached from TP (red dotted circle). **D**, Detachment of both the biceps femoris (BF) and lateral collateral ligament (LCL). The red dashed line represents the joint line, and the blue dotted line represents the front edge of the fibular head. At least two rafting screws were used for adequate gripping of the posterolateral quadrant of the tibial plateau (**D**). FH, fibular head; TT, tibial tubercle; GT, Gerdy’s tubercle; FH, fibular head; ITB, iliotibial tract; BT, biceps tendon; FC, femoral condyle; TP, tibial plateau; BF, biceps femoris; LCL, lateral collateral ligament; POP, popliteus; PL, patellar ligament; CPN, common peroneal nerve

After anesthesia induction and with patients in the supine position, an approximately 8–12-cm curved incision was created at the anterolateral portion of the tibia, which did not extend beyond the front edge of the fibular head (Fig. 2 A). The biceps tendon and lateral collateral ligament complex were retracted posterolaterally. Subperiosteal dissection (not extending beyond the posterior edge of the fibular head) was performed with a sharp knife in the interval between the lateral plateau rim and lateral collateral ligament complex (the gap of the supra-fibular head), and a corridor was created to insert the plate (Fig. 2 A, B, C, and D). The meniscotibial ligament was incised from the tibial attachment to gain access to the articular surface. Most parts of the posterolateral plateau articular surface could be exposed. Then, the depressed articular surface was elevated through either a small augmentative cortical window at the metaphyseal area of the anterolateral surface of the proximal tibia or the fractured window between the lateral plateau and anterior rim. The knee joint was placed at approximately 60° of flexion and varus in the internal rotation position. The lateral collateral ligament (LCL) could be retracted posterolaterally to provide a relatively sufficient visualization and manipulation field. Careful attention is needed because it may be damaged if the LCL is excessively pulled.

#### Restoration and fixation

**Fig. 3:**
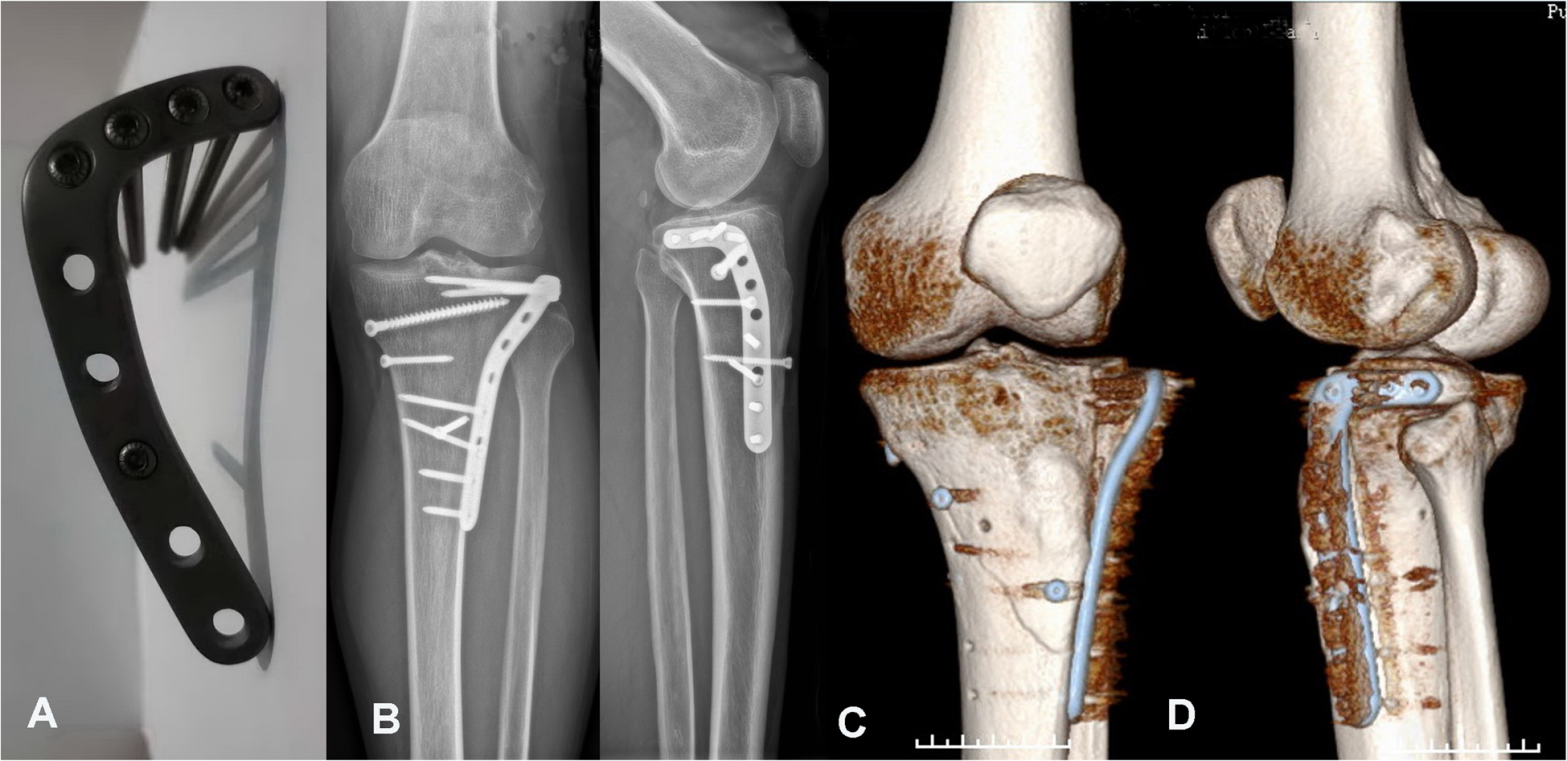
**A**. Our novel plate with a reverse L-shape placed through a modified anterolateral approach. **B**, **C**, and **D**. A patient with a Schatzker classification type V tibial plateau fracture fixed with our plate. **B**. Plain anteroposterior and lateral radiographs obtained post-operatively. **C** and **D**. Three-dimensional computed tomography (CT) reconstruction images obtained post-operatively showing the position of the plate

**Fig. 4:**
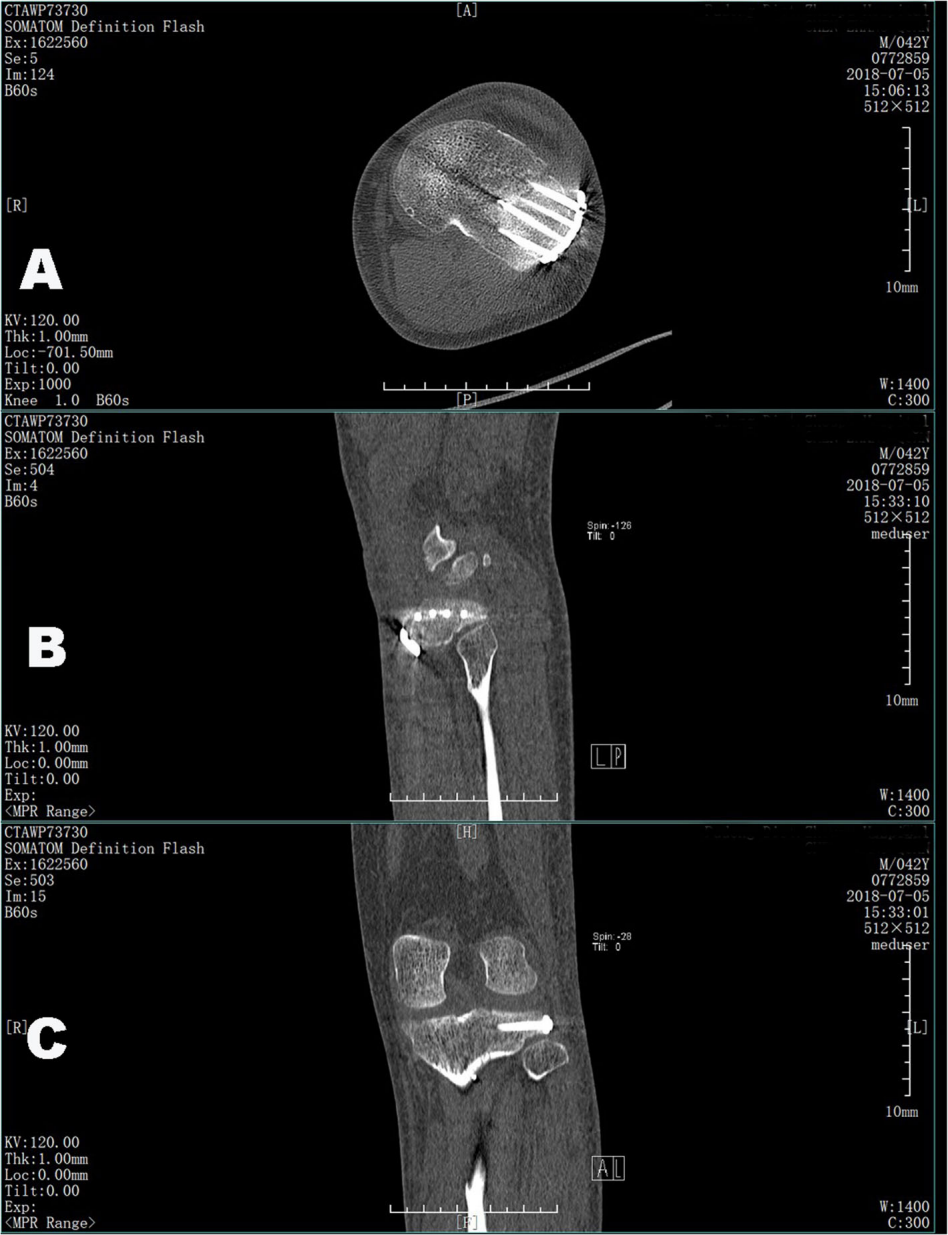
Transverse (**A**), sagittal (**B**), and coronal (**C**) two-dimensional computed tomography (CT) images of the patient in Fig. 3 whose fractured segments were fixed with our plate. At least two rafting screws were used to achieve an adequate grip of the posterolateral quadrant of the tibial plateau (**A** and **B**)

In fact, in most cases of articular rim avulsion of the lateral tibial plateau are at the front edge of the fibular head, the articular fragments are elevated from outside to inside, and the reduced crushed cortex is held in place with multiple K-wires to the lateral epicondyle parallel to the joint line from the medial plateau laterally, so that plate placement is not affected. In cases of large subchondral bone defects, bone void fillers, including autograft, allograft, and commercially available synthetic products (allogenous bone or Osteoset [medical-grade calcium sulfate]; Wright Medical Technology, Memphis, TN, USA) were used. Suppose the fractures involved the distal tibia shaft, our new 3.5-mm lower-profile anatomical lateral proximal tibia locking plate (Fig. 3 A) was inserted in the gap of the supra-fibular head, and the plate was fixed with screws after the C-arm X-ray was performed intra-operatively to assess the reduction and congruence of the articular surface (Fig. 3B-D). Generally, with a longer and narrower transverse arm, this plate can be placed more posteriorly, and at least two posterior screws can penetrate the posterolateral quadrant and achieve raft plate fixation of the lateral condyle (Fig. 4 A-C).

#### Post-operative protocol

Anteroposterior and lateral radiography, as well as CT scans, were performed at 3 days post-operatively to evaluate the articular surface reduction. Patients were supervised while performing active knee joint motion (not exceeding 60°) at 24 h post-operatively for 4 weeks; thereafter, the range of motion (ROM) was increased gradually. Partial weight-bearing with crutches was allowed at 8 weeks post-operatively. Full weight-bearing was not authorized until the bony union was confirmed by radiographs.

#### Assessment

**Fig. 5:**
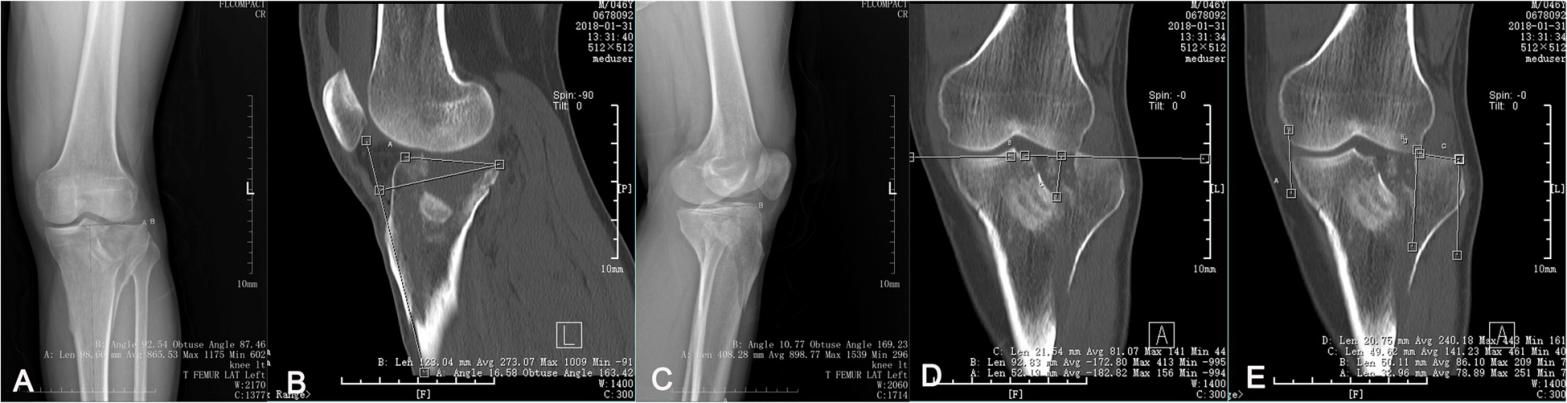
Schematic diagrams showing the method of measuring the tibial plateau-tibial shaft angle (TPTSA), lateral posterior tibial slope angle (LPSTA), step-off, and condylar widening. The anteroposterior view radiograph (**A**) shows that the axial line of the tibial shaft and the tangential line of lateral condyle and medial condyle formed the TPTSA (about 92.54°). The LPSTA is measured on the two-dimensional sagittal CT images of the lateral tibial plateau (about 16.58°). In most cases, the cortex of the posterior wall of the lateral tibial plateau was not collapsed. This method can directly measure the LPSTA of the lateral tibial plateau, not the LPSTA of the knee (**C**) (about 10.77°), i.e., the angle between extending the vertical line of the anterior tibial cortex and the line along the tibial plateau represents the LPSTA on the lateral radiograph of the knee. Step-off was measured on two-dimensional coronal-computed tomography images (**D**, about 21.54 mm). Line A is the tangent line of the medial tibial plateau, and line B is parallel to line A. Normally, the lateral tibial plateau is about 2–3 mm higher than the medial tibial plateau; in the fracture here, the articular surface collapsed, but the residual tibial spine can be used as a marker to measure the step-off. Condylar widening (**E**, about 20.75 mm) was measured by first determining the medial margin of the medial tibial plateau and the medial femoral condyle to be in a state of “kissing,” in the largest condylar widening view on two-dimensional coronal-computed tomography images; the greatest articular surface widening of the condylar was measured

Anteroposterior and lateral radiographs, as well as three-dimensional CT reconstruction images of the injured limbs, were obtained on admission. The tibial plateau-tibial shaft angle (TPTSA) (Fig. 5 A), lateral posterior tibial slope angle (LPSTA) (Fig. 5B, C), step-off (Fig. 5D), and condylar widening (Fig. 5E) were measured on the radiological images pre-operatively, 3 days post-operatively, 3 months post-operatively, and at the final follow-up, modified according to previous documents [20], using a Picture Archiving and Communication System. Finally, the radiological Rasmussen score was calculated [21], the Hospital for Special Surgery (HSS) score [22] was assessed at 3 months post-operatively and at the final follow-up.

#### Features of our newly designed anatomical plate

Our newly designed implants can be used to create rafting constructs with multiple screws to provide subchondral support of these segments using the current approach (Fig. 2 C-D and 4 A-C). The plate (patent number: ZL201720150247.0) was used specifically for Schatzker classification types II, III, V, and VI fractures. The plate is made of a titanium alloy and has a reverse L-shaped anatomical structure (thickness, 1.5 mm; width, 10 mm). Four 3.5-mm screw holes are made in the upper arm; the 4.5-mm lower arm can be set to different lengths according to the patient’s needs (Fig. 3B-D).

### Statistical analyses

Statistical analyses were performed using SPSS 21.0 (IBM Corp., Armonk, NY). Continuous descriptive variables are presented as means standard deviations. The Student–Newman–Keuls, multiple comparison tests, was used to compare multiple groups. Dunnett’s T3 test was used to evaluate differences between groups with unequal variances. Significance was set at 0.05 for all analyses.

## Results

**Fig. 6:**
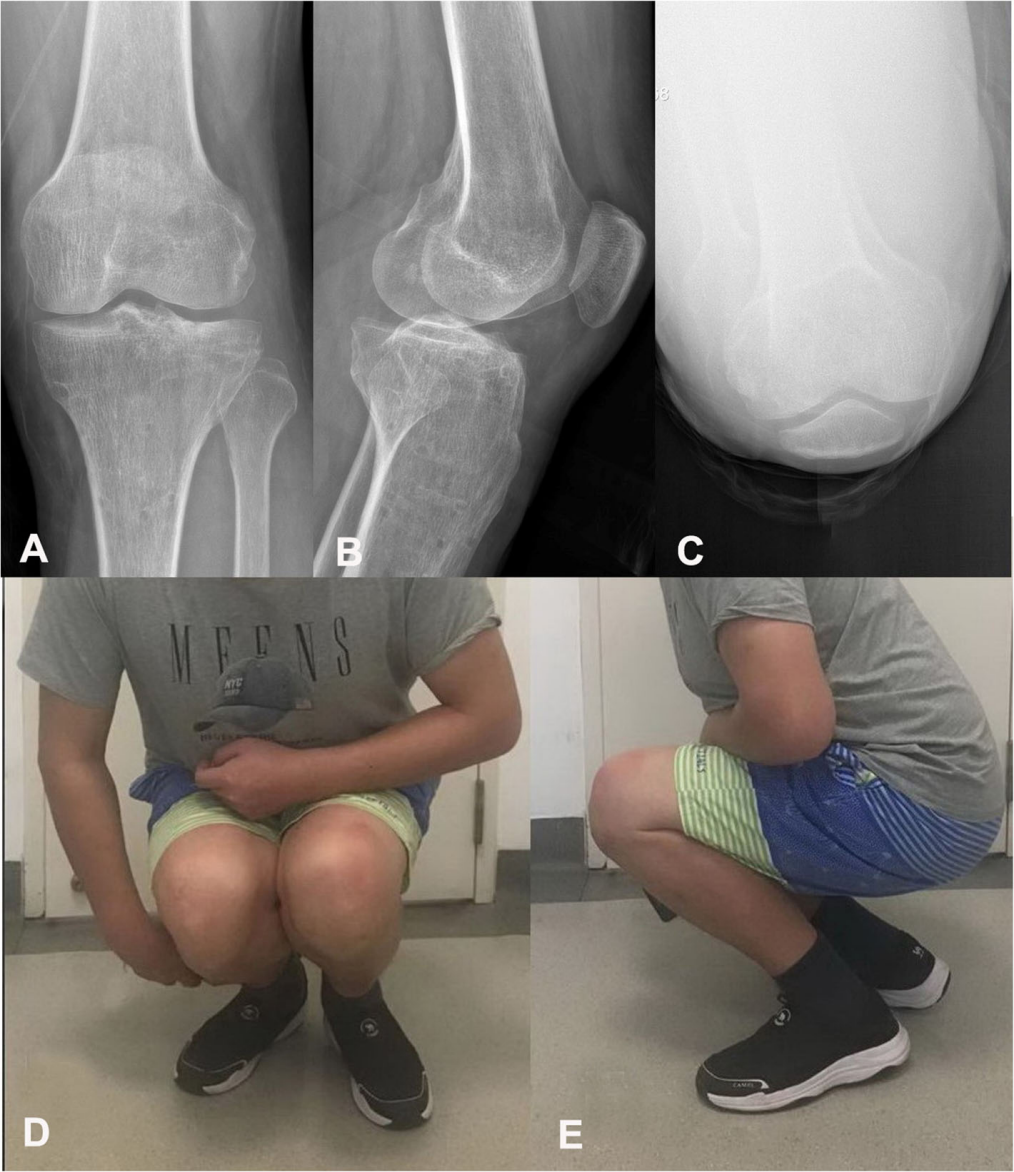
The same patient achieved satisfactory knee joint function at 1 year post-operatively, and his internal fixation was removed (**A**-**E**)

The average follow-up duration was 28.17 ± 7.13 months (range, 17–44 months). All patients achieved bone union after a mean of 12.13 ± 1.36 weeks (range, 9–15 weeks) post-operatively. The TPTSA was 92.24 ± 4.02° at 3 days post-operatively, 90.37 ± 3.41° at 3 months post-operatively, and 90.36 ± 3.41° at the last follow-up, all of which were significantly better than they were pre-operatively (85.14 ± 5.03°) (*p* = 0.001). The LPSTA was 6.56 ± 3.13° at 3 days post-operatively, 6.87 ± 2.44° at 3 months post-operatively, and 6.81 ± 2.40° at the last follow-up, all of which were significantly better than they were pre-operatively (26.42 ± 12.31°) (*p* = 0.001). The step-off was 5.78 ± 2.25 mm at 3 days post-operatively, 5.70 ± 2.35 mm at 3 months post-operatively, and 6.00 ± 2.21 mm at the final follow-up, all of which were significantly lower than they were pre-operatively (20.24 ± 12.45 mm) (*p* = 0.001). The condylar widening was 5.74 ± 2.73 mm at 3 days post-operatively, 5.80 ± 2.77 mm at 3 months post-operatively, and 5.78 ± 2.75 mm at the final follow-up, all of which were significantly smaller than they were pre-operatively (9.49 ± 4.96 mm) (*p* = 0.001). This is also shown by the radiological Rasmussen score results, indicating satisfactory lower limb alignment restoration and no significant reduction loss (Fig. 6 A-E), as shown by comparing the pre-operative (5.91 ± 2.37; range, 2–10) and post-operative radiological Rasmussen scores (9.22 ± 1.78; range, 6–12; 8.96 ± 1.80; range, 6–12 and 9.13 ± 1.69; range, 6–12 respectively) (*p* = 0.001). The HSS score at the final follow-up was 89.10 ± 5.94(range, 78–98), which was significantly higher than that at 3 months post-operatively (84.36 ± 6.76; range, 74–96) (*p* = 0.001), these HSS score results show a gradual resumption of the knee function (Table 2).

**Table 2:** Radiological and Clinical Results at Different Follow-up Periods

Parameters	Pre-operation	Post-operation	3 month-follow up	Last-follow up
**TPTSA (°)**	85.14 ± 5.03^***^ (75.89–95.90)	92.24 ± 4.02 (85.30-98.25)	90.37 ± 3.41 (84.21–97.82)	90.36 ± 3.41 (84.71–97.79)
**LPTSA (**°**)**	26.42 ± 12.31^***^	6.56 ± 3.13	6.87 ± 2.44	6.81 ± 2.40
**Step-off (mm)**	20.24 ± 12.45^***^	5.78 ± 2.25	5.70 ± 2.35	6.00 ± 2.21
**Condylar widening (mm)**	9.49 ± 4.96^***^	5.74 ± 2.73	5.80 ± 2.77	5.78 ± 2.75
**The radiological Rasmussen** **score (range)**	5.91 ± 2.37^***^ (2–10)	9.22 ± 1.78 (6–12)	8.96 ± 1.80 (6–12)	9.13 ± 1.69 (6–12)
**HSS score** **(range)**			84.36 ± 6.76^***^ (74–96)	89.10 ± 5.94 (78–98)

^***^ indicates *p* < 0.001. *TPTSA* tibial plateau-tibial shaft angle; *LPTSA* lateral posterior tibial slope angle; *HSS* Hospital for Special Surgery

No wound infection or common peroneal nerve-related complications were observed, and no LCL lesion was found. No posterolateral instability during flexion occurred at the final follow-up. The axial CT scan demonstrated the trajectory of the screws that gripped the articular fragments from the rim plate (Fig. 3B-D, and 4 A-C). After the fractures healed about 1 year later, the patients had painless knee function, normal ROM, and good clinical outcomes (Fig. 6 A-E).

## Discussion

Theoretically, the optimal approach to the posterolateral quadrant fracture of the tibial plateau should provide maximum visualization with access to perform fracture reduction with rigid fixation while causing minimal damage to the surrounding structures. Currently, numerous approaches have been proposed [3–5, 23], including the posterior approach, posterolateral approach with or without fibular osteotomy, posteromedial approach, and modified anterolateral approach. Posterior and posterolateral approaches offer the distinct advantages of allowing the visualization and manipulation of posterolateral fracture fragments and the posterior application of implants for definitive fixation with posterior buttress or anti-glide plates, thereby allowing early knee motion. However, the mean distance between the lateral tibial plateau and anterior tibial artery penetration point at the interosseous membrane was approximately 46 mm [24]. High variants of the anterior tibial artery should not be ignored because the distance may be < 46 mm in many cases [25]. Furthermore, neither the posterior approach nor the posterolateral approach could be used to manage anterolateral quadrant fractures of the tibial plateau because most cases were accompanied by split wedge fragments of the lateral tibial plateau, including Schatzker classification types II, V, and VI. Other complications were associated with anatomy, large injuries, and prolonged learning curves.

Most orthopedic surgeons are familiar with the conventional anterolateral approach and is used to treat lateral tibial plateau fractures. However, it does not directly expose or allow easy maneuvering of the posterolateral quadrant of the tibial plateau. To overcome these problems, different forms of osteotomy were developed to improve access to the posterolateral quadrant of the tibial plateau and to avoid the hindrance caused by the fibular head and fibular collateral ligaments [26–28]. However, with more serious trauma and increased risks of complications, their clinical application is compromised. The trans-supra-fibular-head approach/modified anterolateral approach used here resulted in minor trauma and easier application without requiring osteotomy.

In contrast to the direct posterolateral and conventional anterolateral approaches, the anterolateral supra-fibular-head approach can provide a safer interval around the rim of the lateral tibial plateau, and fracture reduction could be performed under direct visualization or through the anterolateral cortical window. The relative simplicity of this approach, its ease of positioning, and decreased likelihood of iatrogenic injury to the neurovascular structures are important advantages. Hu et al. performed the anterolateral supra-fibular-head approach and fixation with a lateral raft plate for the posterolateral tibial plateau fracture in seven cases [10]. More recently, they reported a series of 12 isolated posterolateral plateau fractures; the results of both studies show satisfactory HHS scores [12]. It should be noted that the plates used in those studies may not have been specifically designed to treat this type of fracture and may not have allowed more posterior placement of the plate. Meanwhile, their small cohort and retrospective study design could have influenced their conclusions.

It is still unknown whether an anterolateral locking plate can provide sufficient stability to the posterolateral fragment. One study showed that using only one locking screw does not allow adequate gripping of the posterolateral fragment and subsequently leads to fixation failure [13]. Another report showed that 42 % of the lateral tibial plateau remains unsupported after inserting the common anterolateral periarticular plate [29]. Another disadvantage is that it fails to provide stable fixation with classical lateral tibial buttress plates. The distance between the tibial plateau surface and top of the fibular head is usually shorter. It cannot sufficiently accommodate the existing plates, thereby preventing posterior placement of the plate. Plates were not anatomically matched and required pre-contouring, which may increase ligament tension and impede the function recovery of the lateral collateral ligament complex post-operatively. To solve these problems, the authors designed a novel lateral anatomical plate with a long, narrow, transverse ring arm comprising four holes (Fig. 2 C-D and 3 A). The radiographic results of our patients indicated that our newly designed plate could provide adequate fixation of the posterolateral quadrant fractures of the tibial plateau. A lateral buttress plate can provide adequate stability of the fractured fragments, especially in cases of split wedge fragments of the lateral tibial plateau in the sagittal position. Some straightforward techniques had been noted, which can get direct reduction and stable fixation of extended lateral column tibial plateau fractures via a single lateral approach. and a limited tibia condyle osteotomy with a diverging variable angle-LCP (VA-LCP) combined with free subchondral locking screws with a good radiological and fair functional outcome[30]. Some minimally invasive techniques have been developed to treat posterolateral quadrant fractures of the tibial plateau. These include the three-screw jail technique that prevents screw cut-outs through the cancellous bone more than the conventional two-screw osteosynthesis technique[31]. In addition, free subchondral locking screws combined with VA-LCP tend to strengthen structural properties in reconstructing lateral tibial plateau fractures[31]. Our arthroscopically assisted reduction and fixation with screws also satisfied both clinical and radiological outcomes [32].

The main limitations of the present study were the limited number of analyzed patients, relatively short follow-up period, and lack of a control group. Whether posterolateral fracture morphology and the fracture was involved in the posterolateral corner, which is an important consideration for the surgical treatment of this type of fracture, was missed here [33]. Missing information on associated soft tissue injuries, usually evaluated by preoperative magnetic resonance imaging (MRI) and intraoperative examination, was another drawback [34]. Because clinical detection of these lesions in the acute phase of the trauma can somehow be difficult due to pain and swelling [35]. Meanwhile, not all of them require surgery at stage I [36]. Future studies to assess more cases with a longer follow-up duration with a control group are needed to assess the ultimate functional outcomes of the knee joints of such patients.

## Conclusions

Our newly designed anatomical plate placed via the trans-supra-fibular approach successfully exposed and restored the fractured fragments with minor trauma and allowed the surgical procedure to be performed easily without requiring osteotomy. The plate comprises a long, narrow, transverse ring arm and can be used to treat posterolateral tibial plateau fractures by providing at least two holes to affix the posterolateral fracture fragments, resulting in satisfactory clinical outcomes.

Not applicable.

## Data Availability

The data sets supporting the results of this article are included within the article. The datasets are available from the corresponding author on reasonable request.

## Abbreviations

TPTSA: Tibial plateau-tibial shaft angle
LPSTA: Lateral posterior tibial slope angle
HSS: Hospital for Special Surgery
LCP: Locking compression plates
CT: Computed tomography
ROM: Range of motion
VA-LCP: Variable angle-LCP
